# Attentional adjustment in priming tasks: control strategies depend on context

**DOI:** 10.1007/s10339-022-01117-x

**Published:** 2022-12-20

**Authors:** Miriam Tomat, Mike Wendt, Thomas Jacobsen

**Affiliations:** 1 Experimental Psychology Unit, Helmut-Schmidt-University/University of the Federal Armed Forces Hamburg, Holstenhofweg 85, 22043 Hamburg, Germany; 2 Medical School Hamburg, Hamburg, Germany

**Keywords:** Cognitive control, Congruency sequence effect, Attentional adjustment

## Abstract

Goal-directed behavior is assumed to require processes of attentional biasing to counter unwanted action tendencies elicited by distracting stimulus information. This is particularly so if stimulus categories that define the target and the distractor frequently reverse, requiring participants to respond to previously ignored stimulus categories and vice versa. In the current study, we investigated control strategies under such conditions. Specifically, we assessed trial-to-trial modulation of distractor-interference (i.e., congruency sequence effect, CSE) in a temporal flanker task associated with repetition versus alternation of the assignment of stimulus category (i.e., digits, letters) to targets and distractors (i.e., the character presented second or first, respectively) under conditions of a long SOA of 1000 ms (Experiment 1A) and 1200 ms (Experiment 1B). Whereas previous research, using a shorter SOA, suggested temporal-order control (i.e., the occurrence of a CSE in both repetition and—albeit less pronounced—alternation trials), lengthening the distractor-target SOA resulted in a CSE confined to repetition trials, suggesting strong or exclusive reliance on stimulus categories for attentional control (Experiment 1A and B). Adding a redundant stimulus feature (i.e., color), discriminating targets and distractors, eliminated the difference of CSE patterns in repetition and alternation trials (Experiment 2). Together, our results suggest that the strength of concurrently applied control strategies or the choice of a particular control strategy depend on contextual factors.

Control of selective attention is often investigated by means of conflict task protocols. In such protocols, the participant is instructed to respond to task-relevant stimulus information while ignoring task-irrelevant stimulus information. For example, in the well-known Eriksen flanker task (B. A. Eriksen and Eriksen 1974), the participants are instructed to respond to a centrally presented letter (i.e., target) while ignoring the letters surrounding the central letter (i.e., distractors). The distractors and the target are either associated with the same response (e.g., “HHH”—congruent trial) or associated with different responses (e.g., “HSH”—incongruent trial). A typical finding observed in such conflict protocols is increased reaction times (RTs) and higher error rates (ERs) for incongruent trials relative to congruent trials—the *congruency effect*. The congruency effect has been attributed to the co-activation of the distractor-related response in addition to the target-related response in incongruent trials, resulting in response competition (often referred to as *conflict*) (B. A. Eriksen and Eriksen 1974; for an overview, see C. W. Eriksen 1995).

Many studies found that the congruency effect decreases following an incongruent trial compared to a congruent trial; a result pattern referred to as the Gratton Effect or the *congruency sequence effect* (CSE; e.g., Gratton et al. 1992; Schmidt and Weissman 2014; Weissman et al. 2014). The CSE has been attributed to a decrease of conflict in incongruent trials preceded by incongruent trials, possibly resulting from an increase of attentional focusing on target stimulus information (Botvinick et al. 2001, 2004). Among proponents of this attentional adjustment account, there is a controversy about whether to conceive of the adjustment as a form of persistence from the preceding trial (i.e., carry-over of stronger attentional control implemented in incongruent than in congruent trials, e.g., Botvinick et al. 2001; Scherbaum et al. 2011), preparation for a following trial (based on expectations concerning a repetition of the congruency level, e.g., Erb and Aschenbrenner 2019), or episodic retrieval of a previously adopted attentional set when encountering a corresponding congruency level (e.g., Spapé and Hommel 2008). Alternative accounts have been put forward, which relate the CSE to confounds of congruency levels’ sequence with repetitions of conjunctions of low-level stimulus features and responses (for an overview, see Schmidt 2013). On the other hand, recent developments of "confound-minimized protocols,” precluding repetitions of critical low-level features, demonstrated that the CSE could often be observed even without such confounds (for an overview, see Braem et al. 2019).

Although various conflict tasks and stimulus presentation procedures have been used to investigate the CSE, priming protocols, in which a distractor and a target stimulus object are presented successively, received particular attention in recent years. Empirically, the CSE has consistently been observed in such situations (e.g., Hazeltine et al. 2011; Schmidt and Weissman 2014; Tomat et al. 2020, 2021)—more robust than in other confound-minimized conflict tasks (Weissman et al. 2014). Methodologically, successive stimulus presentation has additional advantages, however, such as allowing the investigation of distractor processing before target onset by physiological measures. For instance, Jost et al. (2017, 2019) applied EEG recording to a temporal flanker task (in which discriminating targets and distractors are drawn from the same set of stimuli, Hazeltine et al. 2011) and varied the length of the stimulus onset asynchrony (SOA). Analyzing the Lateralized readiness, potential provided evidence concerning the time course of activation of the response associated with the distractor before target onset (cf. Bartholow et al. 2011).

To account for the occurrence of a CSE in situations of successive presentation of distractors and targets, it seems straightforward to assume that participants prioritize target over distractor information by applying more attentional weight at the time of target presentation than at the time of distractor presentation. Mechanisms of such allocation of attention to temporal positions have been investigated in temporal orienting tasks. For instance, Coull and Nobre (1998) presented cues in advance of target stimuli, contingent with the length of a foreperiod, and found faster responses if the cue was valid compared to invalid (for an overview, see Nobre and van Ede 2018). Assuming a more biased application of attentional weights to temporal positions after incongruent than after congruent trials would explain the CSE. We refer to the mechanism of implementing a stronger bias in the distribution of attentional weights across the temporal positions of target and distractor occurrence as *temporal order control*.

Inferring that temporal order control is applied in priming procedures from observations of a CSE might be questioned, however, for at least two reasons. First, target and distractor stimuli in typical priming procedures previously used tend to be associated with additional discriminative features, such as size (e.g., Schmidt and Weissman 2014; Weissman et al. 2015), allowing for the possibility that attentional selection is based on these features rather than on temporal order. Second, even if no additional discriminative features are present—as is the case in the “temporal flanker task” introduced by Hazeltine et al. (2011)—various confounds with sequences of low-level features and distractor-related contingencies need to be controlled to preclude attributing the CSE to non-attentional processes (see Braem et al. 2019, for an overview, and see Tomat et al. 2020, 2021, for discussion of these issues, focusing on priming procedures).

However, two recent studies provided sophisticated evidence of temporal order control as the source of the CSE in priming procedures. First, Dignath et al. (2021) administered a confound-minimized protocol, including random administration of trials in which the distractor occurred on the first and the target on the second temporal position and trials with reversed presentation order. This was achieved by drawing distractors and targets from different perceptual categories (i.e., arrows vs. letters), thus using the stimulus categories to indicate targets and distractors independently of presentation order. Although trials in which the target was presented before the distractor yielded a generally lower congruency effect than trials in which the distractor preceded the target, reliable CSEs were observed for both types of trials, but only when the presentation order was repeated from the preceding trial. Additional evidence for temporal order control comes from a temporal flanker task study by Tomat et al. (2021). In that study, the distractor was presented first, and the target was presented second throughout the whole experimental session. On each trial, the target and distractor were drawn from different stimulus categories (i.e., digits and letters), and the assignment of stimulus category to the target and the distractor varied randomly (i.e., a target digit could follow a letter distractor or a letter target could follow a digit distractor). With this setup, attentional selection could theoretically be based on stimulus category rather than on temporal position. Persistence of the attentional set to the following trial (or preparatory adoption thereof) would be expected, however, to yield a CSE only in trials in which the assignment of target and distractor to digits and letters repeated. Evidence for temporal order control was seen in the occurrence of a CSE not only in trials involving repetition of the order of stimulus categories but also in trials in which this assignment alternated (e.g., a trial involving a distractor digit and a target letter preceded by a trial involving a distractor letter and a target digit).

Despite this evidence for temporal order control, however, Tomat et al. (2021) also observed a tendency (significant in Experiments 2 and 3) for a larger CSE in stimulus category-order repetition than in category-order alternation trials, a result pattern consistent with the assumption of combined effects of temporal order control and control applied to the processing of different stimulus categories. Thus, whereas Dignath et al. found no evidence for feature-based control (i.e., absence of the CSE in order-alternation trials), the reduction of the CSE in category-alternation trials in Tomat et al.’s (2021) study is consistent with an additional category- or feature-based control mechanism working alongside with temporal order control. Together, the results of the two studies suggest a context-dependent selection of control strategies in priming procedures. Another piece of evidence consistent with the notion of the application of multiple control strategies was obtained by Weissman et al. (2015; Experiments 1 and 2). Specifically, comparing simultaneous and sequential distractor-target presentation procedures, these authors observed a larger CSE in the latter than in the former. As Dignath et al. (2021) pointed out, because distractors were consistently larger than targets in that study, this finding might be explained by assuming the combined work of control based on the temporal order of stimulus presentation with control based on perceptual features, yielding a larger effect in the sequential condition than in the simultaneous condition, in which temporal order control could not be applied.

Another aspect of the study of Weissman et al. (2015) is of interest regarding the strategies of selection applied in priming protocols. Precisely, no difference in the size of the CSE was found when comparing conditions of a short SOA of 166 ms and a longer one of 1133 ms (Weissman et al. 2015, Experiment 3). However, the overall congruency effect (averaged across trials following congruent and incongruent predecessor trials) was effectively eliminated in the latter condition^1^, and the congruency effect following an incongruent trial was reversed (i.e., faster responses in incongruent than in congruent trials). While this finding suggests that temporal order control does not substantially differ for long and short SOAs, it strikingly challenges all accounts attributing the CSE to increased attentional focusing after incongruent trials. This is because enhanced attentional focusing (such as a stronger bias of attentional weights allocated to the first and the second temporal position of stimulus occurrence) should, at the most, result in the absence of an influence of the distractor on behavior. In line with this reasoning, Weissman et al. (2015) favored a response modulation account, which attributes the CSE to increased inhibition of distractor-related response activation after incongruent trials (Ridderinkhof 2002).

However, based on Weissman et al.’s (2015) study, questioning the attentional adjustment account of the CSE, in general, could be premature because their result may be bound to the particular type of response selection demands applied in that study. Precisely, the use of a two-choice task rendered it impossible to preclude the application of a priming-reversal strategy (i.e., distractor-based activation of the other response than the one assigned to the stimulus by instruction after incongruent trials), which might have caused or at least contributed to the reversal of the congruency effect after incongruent trials (cf. Tomat et al. 2020; Wühr and Kunde 2008). Another caveat associated with the interpretation of the reversal of the congruency effect after incongruent trials in Weissman et al. (2015) concerns the confound of temporal order and stimulus size (i.e., targets were smaller than distractors), allowing for both temporal order control and feature-based control.

In light of the open questions regarding the mechanism underlying the CSE in a priming task with long SOA and the potential of the task developed by Tomat et al. (2021) for providing evidence of the application of multiple control strategies, the current study aimed at investigating the interplay of temporal order control and feature- or category-based control under conditions of long distractor-target SOA. To this end, we replicated Experiment 2 of Tomat et al. (2021) (i.e., four-choice temporal flanker task with varying order of stimulus categories assigned to the target and the distractor on a trial, allowing for a confound-minimizing data analysis), replacing the short SOA used in that study with a long SOA of 1000 ms. As laid out above, observing a larger CSE in category-order repetition trials than in category-order alternation trials would be consistent with the application of multiple control strategies (one based on temporal order and one based on stimulus category). Furthermore, as our task involved a four-choice decision, a reversal of the congruency effect after incongruent trials could not be attributed to a priming reversal strategy and would thus strongly corroborate doubts concerning the attentional adjustment account of the CSE.

## Experiment 1A

### Method

***Participants.*** The sample size was determined by a comparable experiment (main differences to Experiment 1: 12 blocks of 96 trials, each one-third of which was associated with another task; see (Tomat et al. 2021), for which the RT analysis yielded a substantial CSE with 16 participants (*η*_*p*_^2^ = 0.18). Seventeen healthy students (eight females and nine males) ranging in age from 20 to 29 years of the Helmut-Schmidt-University/University of the Federal Armed Forces Hamburg gave informed consent to participate in a single-session study in exchange for partial fulfillment of course requirements.

***Stimuli.*** Stimuli material for the temporal flanker task was the letter-digit pairs A-1; B-2; C-3; D-4. The stimuli measures were 8 mm × 13 mm, subtending approximately 0.66° horizontally and 1.10° vertically; and the frame measured 41 mm × 43 mm, subtending approximately 3.36° horizontally and 3.52° vertically. All stimuli were presented in the screen center, white on a grey background.

**Fig. 1:**
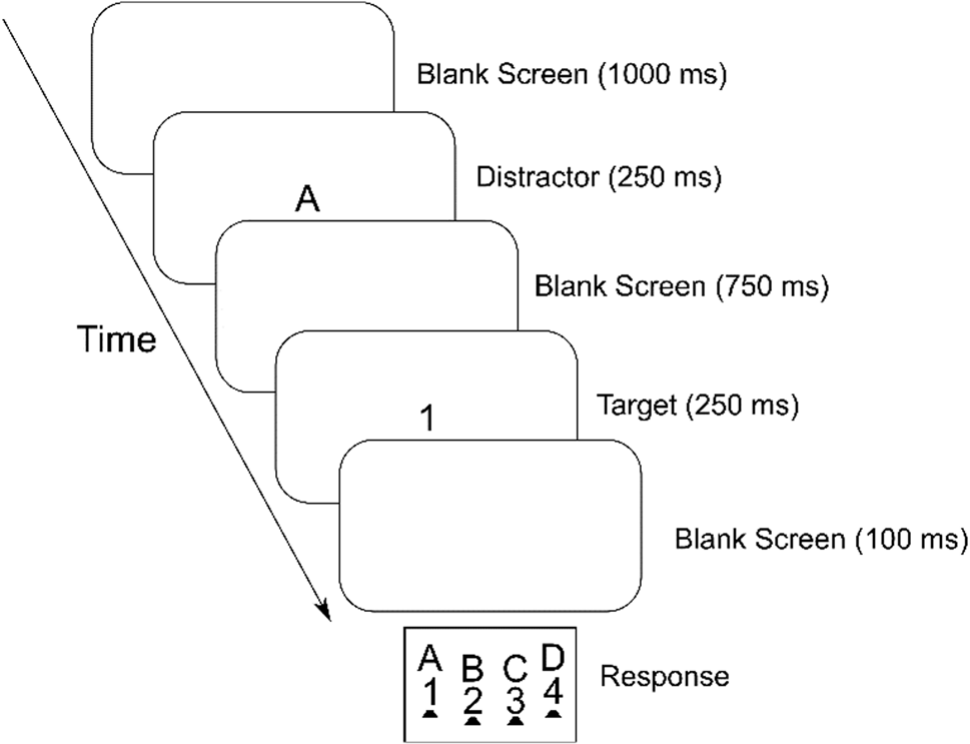
Schematic diagram of a (congruent) temporal flanker task trial involving the successive presentation of a distractor letter and a target digit

***Procedure.*** Participants viewed the screen (refresh rate of 60 Hz) from a distance of about 70 cm. Each trial of the temporal flanker task involved the consecutive presentation of a distractor stimulus and a target stimulus, each shown for 250 ms and separated by a blank interval of 750 ms (see Fig. 1). Thus, the distractor–target SOA was 1000 ms. Following an incorrect response, a message “falsch” (German for incorrect) was presented for 800 ms right below the stimulus frame. For every trial, distractor and target were chosen randomly with the constraint that distractor and target were a digit and a letter or vice versa. Responses were collected using a purpose-built keyboard (response time resolution < 1 ms). The letters were mapped onto the response keys in alphabetical order from left to right, and the digits were mapped onto the response keys in numerical order from left to right. Participants pressed the four lateral keys with the middle and index fingers of their left and right hands and were instructed to rest their fingers on the keys between trials. Blocks of 96 trials were presented, starting with a practice block followed by overall 18 experimental blocks with 96 trials per block. For a schematic diagram of a temporal flanker task trial, see Fig. 1.

***Data analysis.*** In the following analyses, we excluded data from trials associated with RTs below 200 ms or above 2500 ms. Only data from trials involving a correct response were subjected to the RT analyses. We also excluded data from the first three trials per block, from the practice block, and trials following an error. Finally, the analyses were confined to data from trials devoid of feature repetitions and repetitions of the assigned response. 29% of trials were excluded in total. We filtered the data with the *tidyverse* package (Wickham 2017) and conducted the analysis with the *ez* package (Lawrence 2016) implemented in R (R Core Team 2017).

Analyses of variance (ANOVAs) with repeated measures on the factors Congruency Level of Current Trial (congruent vs. incongruent), Congruency Level of Preceding Trial (congruent_*n*−1_ vs. incongruent_*n−*1_), and Sequence of Target/Distractor Category (repetition vs. alternation) were conducted on the RTs and error proportions. The planned comparisons (repeated measures ANOVAs) were conducted on repetition and alternation of Sequences of Target/Distractor Category trials mean RTs and error proportions separately. Note that we report the results of one-tailed significance tests due to our directional hypotheses.

### Results

***RT.*** The overall ANOVA yielded a significant congruency effect of Congruency Level of Current Trial; *F*(1,16) = 9.87, *p* = 0.003, *η*_*p*_^2^ = 0.38). The main effect of Congruency Level of Preceding Trial also reached significance (*F*(1, 16) = 16.37, *p* < 0.001, *η*_*p*_^2^ = 0.51) as did the main effect of Sequence of Target/Distractor Category (*F*(1, 16) = 14.67, *p* < 0.001, *η*_*p*_^2^ = 0.48). More importantly, a significant two-way interaction, between Congruency Level of Current Trial and Congruency Level of Preceding Trial reached significance (*F*(1, 16) = 4.44, *p* = 0.003, *η*_*p*_^2^ = 0.22), indicating a CSE. This was, however, qualified by a three-way interaction with Sequence of Target/Distractor Category (*F*(1, 16) = 10.9, *p* = 0.002, *η*_*p*_^2^ = 0.4). Furthermore, the two-way interaction between Congruency Level of Current Trial and Sequence of Target/Distractor Category reached significance (*F*(1, 16) = 4.44, *p* = 0.003, *η*_*p*_^2^ = 0.22), reflecting that the congruency effect was decreased for repetition relative to alternation trials. The two-way interactions between Congruency Level of Current Trial and Congruency Level of Preceding Trial, and between Congruency Level of Preceding Trial and Sequence of Target/Distractor Category did not reach significance (*F*(1, 16) = 0.45, *p* = 0.26, *η*_*p*_^2^ = 0.03; *F*(1, 16) = 0.17, *p* = 0.34, *η*_*p*_^2^ = 0.22).

**Fig. 2:**
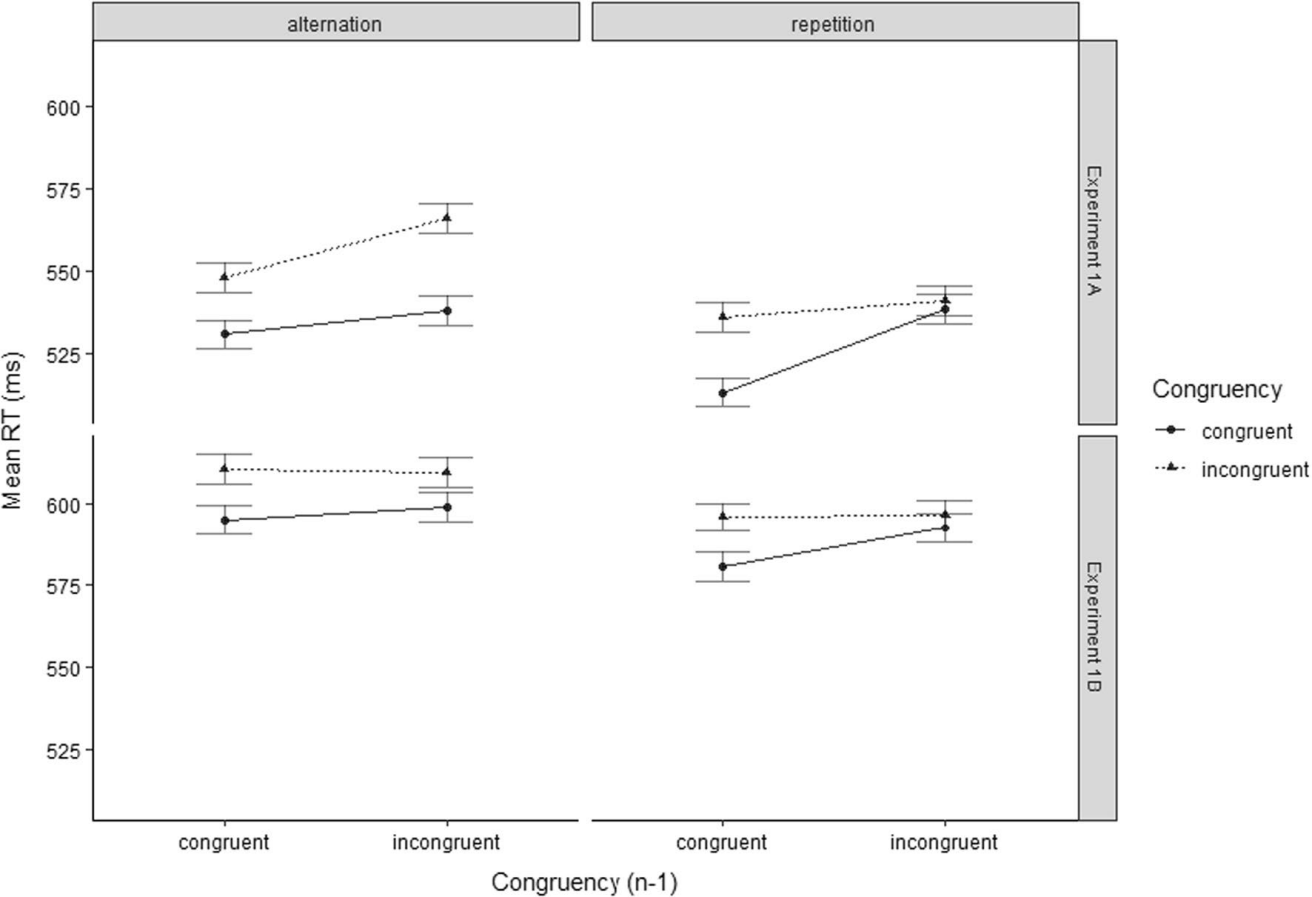
Mean RTs of temporal flanker task trials in Experiment 1A (top), and Experiment 1B (bottom). Data are shown as a function of Congruency Level of Current Trial (congruent vs. incongruent), Congruency Level of Preceding Trial (congruent_*n*−1_ vs. incongruent_*n*−1_), Sequence of Target/Distractor Category (alternation vs. repetition), and Experiment (1A vs. 1B)

The analysis of the trials only involving target/distractor category repetitions revealed a significant congruency effect (*F*(1, 16) = 4.2, *p* = 0.029, *η*_*p*_^2^ = 0.21) as well as a significant main effect of Congruency Level of Preceding Trial (*F*(1, 16) = 9.05, *p* = 0.0042, *η*_*p*_^2^ = 0.36). Most importantly, the CSE reached significance (*F*(1, 16) = 4.18, *p* = 0.029, *η*_*p*_^2^ = 0.21). Conversely, the analysis of trials involving only target/distractor category alternations yielded a marginally significant tendency for a reversed CSE (*F*(1, 16) = 2.36, *p* = 0.07, *η*_*p*_^2^ = 0.13). Still, the analysis revealed a significant congruency effect (*F*(1, 16) = 14.09, *p* < 0.001, *η*_*p*_^2^ = 0.47) as well as a significant main effect of Congruency Level of Preceding Trial (*F*(1, 16) = 7.14, *p* = 0.0083, *η*_*p*_^2^ = 0.31). For an illustration of the effects, see Fig. 2.

***ER.*** The overall ANOVA yielded a significant three-way interaction involving Congruency Level of Current Trial, Congruency Level of Preceding Trial, and Sequence of Target/Distractor Category (*F*(1, 16) = 4.96, *p* = 0.02, *η*_*p*_^2^ = 0.24). The two-way interaction, between Congruency Level of Current Trial and Congruency Level of Preceding Trial did not reach significance (*F*(1, 16) = 0.90, *p* = 0.18, *η*_*p*_^2^ = 0.05), nor did, the two-way interaction between Congruency Level of Current Trial and Sequence of Target/Distractor Category did not reach significance (*F*(1, 16) = 1.10, *p* = 0.16, *η*_*p*_^2^ = 0.06).

**Fig. 3:**
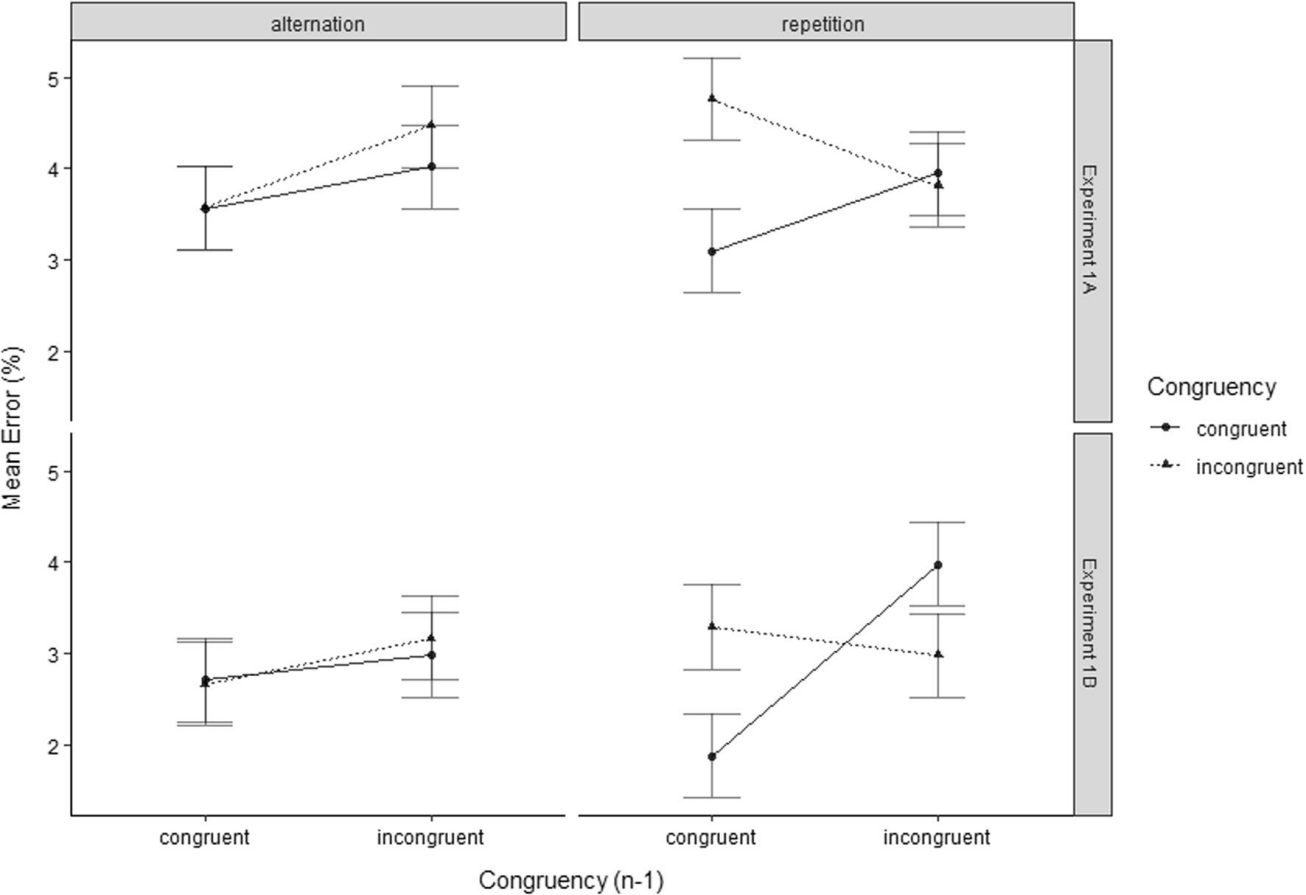
Mean ERs of temporal flanker task trials in Experiment 1A (top), and Experiment 1B (bottom). Data are shown as a function of Congruency Level of Current Trial (congruent vs. incongruent), Congruency Level of Preceding Trial (congruent_*n*−1_ vs. incongruent_*n*−1_), Sequence of Target/Distractor Category (alternation vs. repetition), and Experiment (1A vs. 1B)

The analysis of the trials only involving target/distractor category repetitions revealed a significant congruency effect (*F*(1, 16) = 3.12, *p* = 0.048, *η*_*p*_^2^ = 0.16) as well as a significant CSE (*F*(1, 16) = 3.55, *p* = 0.039, *η*_*p*_^2^ = 0.18). The analysis of trials only involving target/distractor category alternations did not yield a significant congruency effect (*F*(1, 16) = 0.22, *p* = 0.32, *η*_*p*_^2^ = 0.01) nor a significant main effect of Congruency of Preceding Trial (*F*(1, 16) = 2.23, *p* = 0.078, *η*_*p*_^2^ = 0.12) nor a significant CSE (*F*(1, 16) = 0.31, *p* = 0.29, *η*_*p*_^2^ = 0.02). For an illustration of the effects, see Fig. 3.

### Discussion

Administering a four-choice temporal flanker task—thus preventing a priming reversal strategy—under conditions of a long distractor-target SOA of 1000 ms yielded results that differed in relevant aspects from both the findings of Experiment 3 of Weissman et al. (2015) and of the experiments of Tomat et al. (2021). In contrast to Weissman et al. (2015), we observed a significant overall congruency effect and no overall significant CSE. This was because, unlike in the study of Tomat et al. (2021), the CSE was confined to category-order repetition trials. In contrast, category-order alternation trials were associated with a marginally significantly reversed CSE. Based on our reasoning that temporal order control and category-based control should work in the same direction in category repetition trials (i.e., reducing the congruency effect after incongruent trials, thus producing a CSE) but not in category alternation trials, in which temporal order control would, again, produce a CSE but control based on stimulus categories might even work in the opposite direction (for instance, because responding to a target from the category inhibited on the preceding trial might make processing more susceptible to the distractor), the results observed in Experiment 1A might, generally, be accounted for in terms of a change in the relative effects of the two control strategies. Two different versions of this idea appear plausible. The first explanation relates to the previously mentioned observation that the congruency effect steadily decreases when the SOA is increased, possibly resulting in a reversed congruency effect at sufficiently long intervals. To account for this finding, Machado et al. (2007, 2013) suggested that processing the distractor in priming tasks is subject to inhibition during the SOA. Assuming that such progressively increasing inhibition not only pertains to individual stimuli but—at least in a context in which targets and distractors are drawn from discernibly different categories as in our task—generalizes to the whole category of the current distractor would predict larger category-based inhibition in long than in short SOA conditions. Working in the opposite direction than temporal order control in category alternation trials, stronger category-based control may outweigh and possibly exceed the effect of temporal order control, leveling out or even reversing the CSE, respectively. Note that this explanation does not assume a qualitative change in control strategies. In particular, it would be consistent with the notion that lengthening the SOA has no effect on the application of temporal order control.

The results of Experiment 1A could also be accounted for, however, by assuming that lengthening the SOA weakened temporal order control, possibly resulting—as an extreme case—in complete abandoning thereof. This possibility relates to a considerable body of literature discussing the specificity of sequential conflict adjustment to the type of conflict or the distracting information by which conflict is evoked (for overviews, see, e.g., Braem et al. 2014; Egner 2008). Studies in which distractor-target conflict was evoked by varying types of distracting information (such as, for example, Eriksen-type flankers and Stroop-like words) yielded inconsistent results concerning the generalization of the CSE between conflict types. In this connection, Akҫay and Hazeltine (2008) suggested that generalization occurred if the stimulus–response events presented on consecutive trials are represented as a single task, whereas conceiving of them as two different tasks yields a CSE confined to task repetition trials (cf. Lim and Cho 2018). Indeed, task switching studies involving an examination of the CSE repeatedly demonstrated a CSE confined to task repetition trials (Brown et al. 2007; Kiesel et al. 2006; Wendt et al. 2013).

In task switching studies, participants frequently alternate between executing two different tasks (e.g., parity vs. magnitude judgment regarding a stimulus digit). Assigning a response category of each task to each of the response keys (e.g., odd and smaller than 5 to a left-sided key and even and larger than 5 to a right-sided key) results in a subgroup of stimuli affording the same response in both tasks (hence congruent condition, e.g., 1 or 8 in the above example) and another subgroup affording different responses in the two tasks (hence incongruent condition, e.g., 2 or 7). A congruency effect (i.e., faster and more accurate responses in congruent than in incongruent trials) is a typical result obtained in task switching studies (e.g., Meiran 1996; Rogers and Monsell 1995; for an overview, see Kiesel et al. 2006). Intriguingly, the temporal flanker task we used resembles classical task switching procedures in which participants frequently alternate between categorizing a letter or a digit in the presence of a distractor from the other stimulus category (or task) (e.g., Brown et al. 2007; Roger and Monsell 1995). Against this background. Obtaining evidence for temporal order control in short SOA conditions but not in long SOA conditions might be interpreted as indicating that lengthening the SOA yielded a change toward a task-specific control strategy, such as inhibiting the processing of stimuli of the other task, while abandoning temporal order control, consistent with Akҫay and Hazeltine’s (2008) conjecture concerning a shift from a “single-task representation” to a representation of separate tasks.

Although our data do not allow any firm conclusions about whether such a shift occurred, a typical finding obtained in task switching studies (i.e., under conditions assumed to be associated with a “two-tasks representation”) could be of interest here. Specifically, task switching studies usually involve an interaction of the sequence of tasks and the sequence of responses. This is because task switch trials tend to be faster and less error-prone when the required response is different from the response of the previous trial (whereas no such difference or even a response repetition advantage is found in task repetition trials.) Although the precise mechanism underlying this interaction is subject to current discussion (e.g., Druey 2013), it might be considered a tentative indication for a representation of two separate tasks. We, therefore, repeated the analyses of the data of Experiment 1A, including all trials associated with repetitions of stimulus features or responses, and added the sequence of responses (repetition, alternation) as an additional factor. Neither the analysis of RTs nor the analysis of error rates yielded a substantial interaction of Target/Distractor Category Sequence and Response Sequence (*F*(1,16) = 0.04 and 0.26; *p* = 0.42 and 0.06, respectively). We thus obtained no preliminary evidence in favor of a two-task representation.

The regular CSE observed in category-order repetition trials appeared to be associated with the elimination of the congruency effect after incongruent predecessor trials (see Figs. 2 and 3) rather than with the reversal thereof, as reported by Weissman et al. (2015, Experiment 3). At the current stage, we cannot determine whether this discrepancy was brought about by diverting from the use of two-choice tasks—preventing a priming-reversal strategy—or whether a longer SOA than the 1000 ms used in our experiment is needed for the reversal to occur. Thus far, the results obtained in the current experiment clearly do not contradict the attentional adjustment account. Nevertheless, the interaction of the CSE with the sequence of category order is also interesting from the perspective of the response modulation account. As it would seem quite implausible to assume that inhibition of the distractor-related response on category alternation trials is more pronounced after congruent than after incongruent trials, reversal of the CSE (albeit only marginally significant in our experiment) would need a different explanation. How could the response modulation hypothesis account for a reversed CSE in category alternation trials? Resembling the notion of category-based attentional adjustment, adjustment of response inhibition may be linked to the category of the current distractor stimulus. Such a mechanism would impair responding in category alternation trials following an incongruent predecessor trial as the current target comes from the category for which response inhibition was increased. This impairment may, in turn, weaken the target-related response in competition with the response associated with the distractor, thus increasing the congruency effect compared with trials following a congruent predecessor trial.

## Experiment 1B

Although the two explanations we considered for the selective occurrence of a CSE in category repetition trials in Experiment 1A involve substantial differences (one attributing it to a by-product of a change in effectiveness of category-based control in long SOA conditions, the other one assuming a shift in control strategies possibly evoked by a change in task representation) both comprise the notion that multiple control strategies can be applied concurrently and that contextual factors can affect their contribution to task performance. As this notion has considerable theoretical importance, we strived to replicate the relevant finding (i.e., the confinement of the CSE to category repetition trials when the SOA is long).

Also, our attempt to examine the occurrence of a reversed congruency effect after incongruent trials might be considered inconclusive as it cannot be dismissed that administering an even longer SOA could result in the complete elimination of the CSE (that is, our manipulation might have been too weak to allow testing the response modulation hypothesis). We, therefore, replicated Experiment 1A with the only alteration that the SOA was set to an even larger value of 1200 ms.

### Method

***Participants.*** 30 healthy students (10 female and 20 male) ranging in age from 19 to 29 years of the Helmut-Schmidt-University/University of the Federal Armed Forces Hamburg gave informed consent to participate in a single-session study in exchange for partial fulfillment of course requirements.

***Procedure***. Experiment 1B was identical to Experiment 1 in terms of method, except that in Experiment 1B, the SOA was lengthened to 1200 ms.

### Results

***RT.*** The overall ANOVA yielded a significant congruency effect (*F*(1, 29) = 12.53, *p* = 0.0013, *η*_*p*_^2^ = 0.30) as well as a significant main effect of Sequence of Target/Distractor Category (*F*(1, 29) = 28.65, *p* < 0.001, *η*_*p*_^2^ = 0.50. The main effect of Congruency Level of Preceding Trial did not reach significance (*F*(1, 29) = 1.7, *p* = 0.10, *η*_*p*_^2^ = 0.06). Furthermore, the CSE did not reach significance (*F*(1, 29) = 1.87, *p* = 0.09, *η*_*p*_^2^ = 0.06) nor did the two-way interactions between Congruency Level of Current Trial and Sequence of Target/Distractor Category and between Congruency Level of Preceding Trial and Sequence of Target/Distractor Category (*F*(1, 29) = 0.43, *p* = 0.26, *η*_*p*_^2^ = 0.01; *F*(1, 29) = 1.54, *p* = 0.11, *η*_*p*_^2^ = 0.05) nor three-way interaction between Congruency Level of Current Trial with Congruency Level of Preceding Trial and Sequence of Target/Distractor Category (*F*(1, 29) = 0.48, *p* = 0.25, *η*_*p*_^2^ = 0.02).

The analysis of the trials only involving target/distractor category repetitions revealed a significant congruency effect (*F*(1, 29) = 5.83, *p* = 0.011, *η*_*p*_^2^ = 0.17) as well as a significant main effect of Congruency Level of Preceding Trial (*F*(1, 29) = 3.09, *p* = 0.045, *η*_*p*_^2^ = 0.10). The CSE did not reach significance (*F*(1, 29) = 1.90, *p* = 0.09, *η*_*p*_^2^ = 0.06). The analysis of trials involving only target/distractor category alternations yielded a significant congruency effect (*F*(1, 29) = 9.82, *p* = 0.0019, *η*_*p*_^2^ = 0.25). The main effect of Congruency Level of Preceding Trial did not reach significance (*F*(1, 29) = 0.16, *p* = 0.34, *η*_*p*_^2^ = 0.08) nor did the CSE (*F*(1, 29) = 0.42, *p* = 0.21, *η*_*p*_^2^ = 0.01). For an illustration of the effects, see Fig. 2.

***ER*.** The overall ANOVA yielded a significant main effect of Congruency Level of Preceding Trial (*F*(1, 29) = 14.90, *p* < 0.001, *η*_*p*_^2^ = 0.34) as well as a significant CSE (*F*(1, 29) = 8.81, *p* = 0.003, *η*_*p*_^2^ = 0.23). Also, the three-way interaction between the CSE and Sequence of Target/Distractor Category reached significant (*F*(1, 29) = 10.06, *p* = 0.002, *η*_*p*_^2^ = 0.26). The congruency effect did not reach significance (*F*(1, 29) = 0.53, *p* = 0.24, *η*_*p*_^2^ = 0.02) nor did the main effect of Sequence of Target/Distractor Category (*F*(1, 29) = 0.47, *p* = 0.25, *η*_*p*_^2^ = 0.02). The two-way interaction between Congruency on Current Trial and Sequence of Target/Distractor Category did not reach significance (*F*(1, 29) = 0.09, *p* = 0.38, *η*_*p*_^2^ = 0.003) nor did the two-way interaction between Congruency on Preceding Trial and Sequence of Target/Distractor Category (*F*(1, 29) = 1.30, *p* = 0.13, *η*_*p*_^2^ = 0.04).

The analysis of the trials only involving target/distractor category repetitions revealed a significant main effect of Congruency Level of Preceding Trial (*F*(1, 29) = 8.34, *p* = 0.004, *η*_*p*_^2^ = 0.22), and a significant CSE (*F*(1, 29) = 14.55, *p* < 0.001, *η*_*p*_^2^ = 0.33). The congruency effect did not reach significance (*F*(1, 29) = 0.38, *p* = 0.27, *η*_*p*_^2^ = 0.01). The analysis for trials only involving target/distractor category alternations did not yield a significant congruency effect (*F*(1, 29) = 0.08, *p* = 0.39, *η*_*p*_^2^ = 0.003) nor a significant main effect for Congruency on Preceding Trial (*F*(1, 29) = 2.66, *p* = 0.06, *η*_*p*_^2^ = 0.08) nor a significant CSE (*F*(1, 29) = 0.25, *p* = 0.31, *η*_*p*_^2^ = 0.01). For an illustration of the effects, see Fig. 3.

### Discussion

Unlike Experiment 1A, Experiment 1B did not produce a significant CSE in the RT analysis. The error results, however, demonstrated a CSE confined to category repetition trials. Experiment 1B thus replicated the crucial aspect of the results of Experiment 1A, albeit for performance accuracy rather than for response speed. Experiment 1B thus corroborated the crucial aspect of the results of Experiment 1A (i.e., CSE confined to category repetition trials), corroborating our conjecture that lengthening the SOA in a temporal flanker task leads to a shift away from temporal order control or to a control strategy based on stimulus categories, either in a gradual manner (e.g., increased effectiveness of category-based control compared with short SOA conditions) or by abandoning temporal order control altogether.

**Fig. 4:**
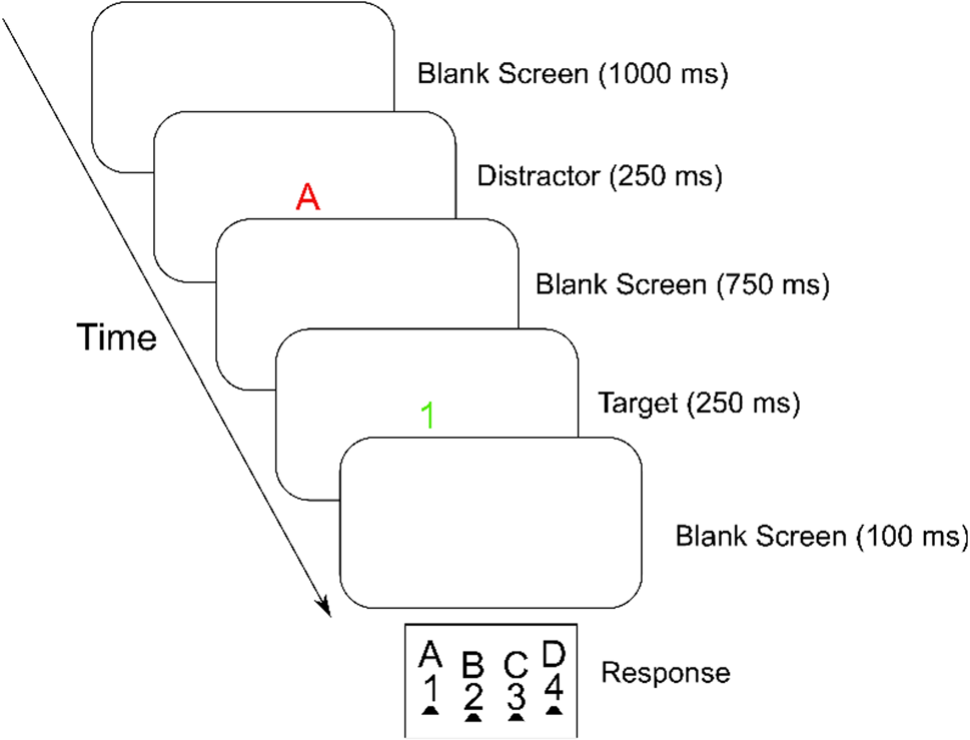
Schematic diagram of a (congruent) temporal flanker task trial involving the successive presentation of a distractor letter and a target digit

A second purpose of Experiment 1B pertained to investigating the occurrence of a reversed congruency effect following incongruent trials, which Weissman et al. (2015) observed under conditions of a long SOA in their Experiment 3. In fact, as regards error rates, the congruency effect in category repetition trials following an incongruent trial was descriptively reversed (see Fig. 4). As regards RTs, however, the congruency effect was still significant overall. Despite the long SOA of 1200 ms. Experiment 1B thus again lacked evidence for a reversal of processing ease (i.e., superior performance in incongruent compared to congruent trials) following incongruent trials, which presents a challenge for theories of attentional adjustment in general.

## Experiment 2

Assuming that the application of one or more particular control strategies in a given task situation depends on contextual factors such as the distractor-target SOA invites further manipulations and assessment of their effects on sequential control effects. Experiment 2 was conducted as a step in this direction. This experiment involved a manipulation that we deemed likely to evoke a category-unspecific control strategy despite the use of a long SOA, a strategy should evidence itself in the form of a CSE in both category repetition and category switch trials. As a means of evoking a category-unspecific control strategy, we added a contextual feature (i.e., stimulus color) that was completely redundant to the temporal position, which defined target- vs. distractorhood.^2^ That is, the stimulus presented first consistently occurred in one particular color, and the stimulus presented second in another one, keeping the color-position assignment constant for the experimental session. We reasoned that, for one thing, such “highlighting of temporal position” might re-install temporal order control by drawing participants’ attention toward the order of stimulus administration as a possible means of target selection. For another, the consistent coloring of targets and distractors could result in using a color-based control strategy, that is, biasing processing in favor of stimuli presented in the “target color,” more so after incongruent than after congruent trials. Although a result pattern involving a CSE in both category repetition and category alternation trials would not allow deciding whether temporal order control or color-based control (or both) was applied, it would demonstrate some form of category-unspecific control under conditions of a long CSE.

### Method

***Participants.*** Sixteen healthy students (eight females and eight males) ranging in age from 20 to 30 years of the Helmut-Schmidt-University/University of the Federal Armed Forces Hamburg participated in a single-session experiment in exchange for partial fulfillment of course requirements. All participants gave written informed consent prior to the experiment.

***Procedure***. Experiment 2 was identical to Experiment 1A in terms of method, except that in Experiment 2, the distractor and target were presented in different colors (red and green, respectively). This color assignment was kept constant throughout the whole experiment but was counterbalanced (half of the participants were presented with the distractor in red and the target in green, while for the other half, it was vice versa). For a schematic diagram of a temporal flanker task trial, see Fig. 4.

***Data analysis.*** The analyses conducted on the data were identical to the analyses conducted in Experiment 1. 30% of trials were excluded in total. Additionally, the same analyses were conducted on both experiments' data with the added between-factor Experiment to compare the effect of the presence versus absence of the redundant color feature.

### Results

**Fig. 5:**
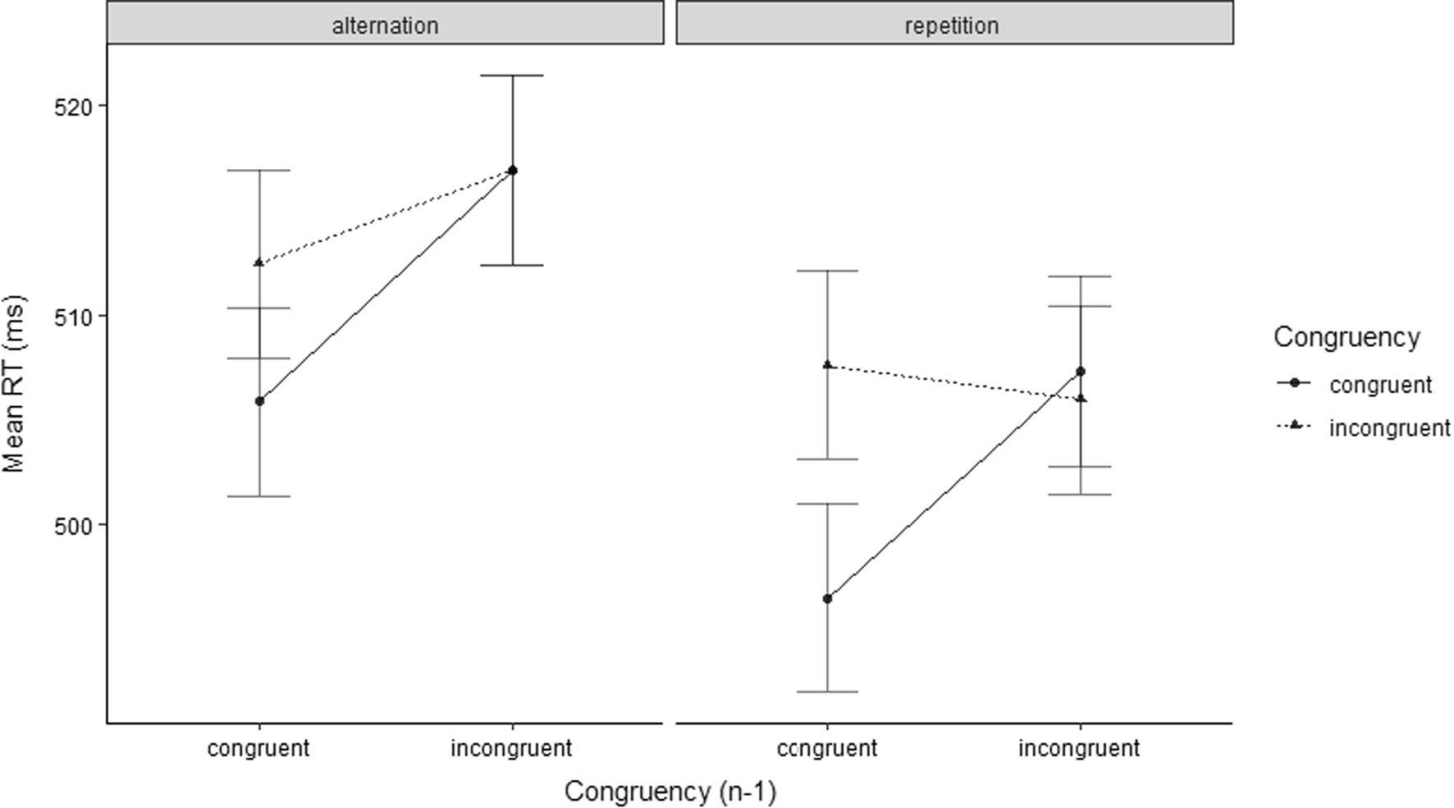
Mean RTs of temporal flanker task trials in Experiment 2. Data are shown as a function of Congruency Level of Current Trial (congruent vs. incongruent), Congruency Level of Preceding Trial (congruent_*n*−1_ vs. incongruent_*n*−1_), and Sequence of Target/Distractor Category (alternation vs. repetition)

**Fig. 6:**
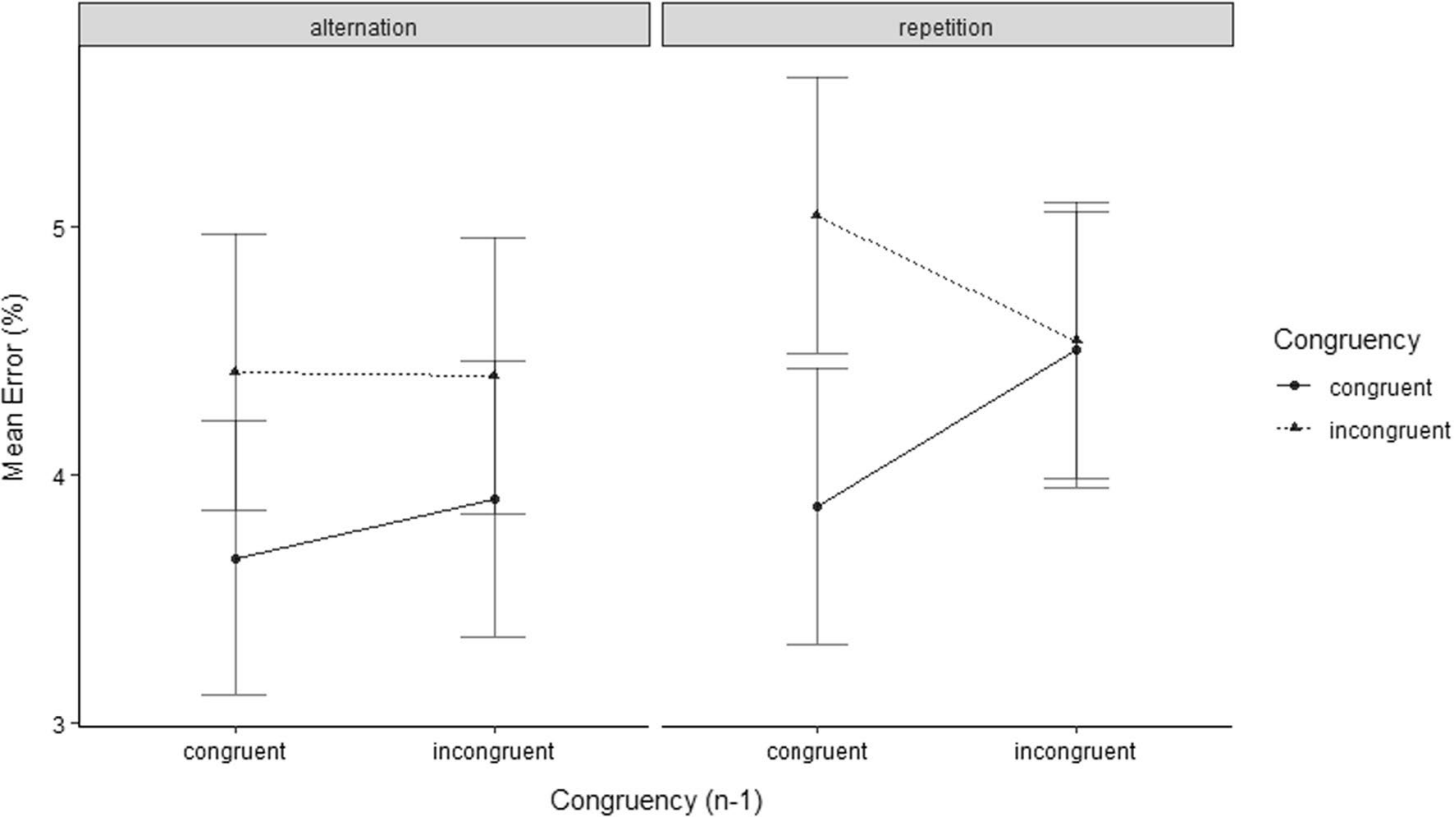
Mean ERs of temporal flanker task trials in Experiment 2. Data are shown as a function of Congruency Level of Current Trial (congruent vs. incongruent), Congruency Level of Preceding Trial (congruent_*n*−1_ vs. incongruent_*n*−1_), and Sequence of Target/Distractor Category (alternation vs. repetition)

***RT.*** See Fig. 5 for the RTs results and Fig. 6 for the ERs results. To ease a comparison with Experiment 1A and 1B see Appendix A and B for a display of the results of all three experiments with the same scaling. The overall ANOVA yielded a marginally significant congruency effect (*F*(1, 15) = 3.03, *p* = 0.051, *η*_*p*_^2^ = 0.17) which is also generally smaller than in Experiment 1A (see Fig. A). The main effect of Congruency Level of Preceding Trial also reached significance (*F*(1, 15) = 5.40, *p* = 0.017, *η*_*p*_^2^ = 0.26) as well as the main effect of Sequence of Target/Distractor Category (*F*(1, 15) = 33.31, *p* < 0.001, *η*_*p*_^2^ = 0.69). More importantly, the ANOVA yielded a significant CSE (*F*(1, 15) = 4.21, *p* = 0.029, *η*_*p*_^2^ = 0.22), which was not modulated by Sequence of Target/Distractor Category (*F*(1, 15) = 0.49, *p* = 0.25, *η*_*p*_^2^ = 0.03). Neither the two-way interaction between Congruency on Current Trial and Sequence of Target/Distractor Category did not reach significance (*F*(1, 15) = 0.02, *p* = 0.31, *η*_*p*_^2^ = 0.02) nor did the two-way interaction between Congruency on Preceding Trial and Sequence of Target/Distractor Category (*F*(1, 15) = 0.49, *p* = 0.24, *η*_*p*_^2^ = 0.03). For an illustration of the effects see Fig. 5.

***ER.*** The overall ANOVA yielded a marginally significant congruency effect (*F*(1, 15) = 2.24, *p* = 0.078, *η*_*p*_^2^ = 0.13). The main effect of Congruency Level of Preceding Trial did not reach significance nor did the main effect of Sequence of Target/Distractor Category (*F*(1, 15) = 0.07, *p* = 0.40, *η*_*p*_^2^ = 0.005; *F*(1, 15) = 1.71, *p* = 0.11, *η*_*p*_^2^ = 0.1). Neither the CSE reached significance nor did the two-way interaction between Congruency Level of Current Trial and Sequence of Target/Distractor Category nor the two-way interaction between Congruency Level of Preceding Trial and Sequence of Target/Distractor Category (*F*(1, 15) = 1.25, *p* = 0.14, *η*_*p*_^2^ = 0.08; *F*(1, 15) = 0.001, *p* = 0.49, *η*_*p*_^2^ = 0.00) (*F*(1, 15) = 0.01, *p* = 0.46, *η*_*p*_^2^ = 0.00). Also the three-way interaction between Congruency Level of Current Trial, Congruency Level of Preceding Trial and Sequence of Target/Distractor Category did not reach significance (*F*(1, 15) = 0.73, *p* = 0.20, *η*_*p*_^2^ = 0.004). For an illustration of the effects, see Fig. 6.

## Comparison Experiment 1A and Experiment 2

As Experiment 1A and Experiment 2 were identical in all respects, with the only exception that targets and distractors were presented in the same color in Experiment 1A and in different colors in Experiment 2, we added an analysis comparing data from both experiments.

***RT.*** Repeating the analyses with Experiment as a between-subjects factor, the overall ANOVA yielded a significant main effect of Congruency Level of Current Trial (*F*(1, 31) = 12.67, *p* = 0.012, *η*_*p*_^2^ = 0.29), a significant main effect of Congruency Level of Preceding Trial *F*(1, 31) = 21.52, *p* < 0.001, *η*_*p*_^2^ = 0.41), and a significant main effect of Sequence of Target/Distractor Category (*F*(1, 31) = 32.59, *p* < 0.001, *η*_*p*_^2^ = 0.51). Also, the three-way interaction involving the CSE and Sequence of Target/Distractor Category reached significance (*F*(1, 31) = 8.79, *p* = 0.0029, *η*_*p*_^2^ = 0.22). Most importantly, the four-way interaction with Experiment was significant (*F*(1, 31) = 3.86, *p* = 0.029, *η*_*p*_^2^ = 0.11). For a complete list of results, see Appendix C.

Since the four-way interaction was significant, we analyzed the data from each sequence of category-order separately. For trials involving category repetition trials, there was a significant congruency effect (*F*(1, 31) = 6.43, *p* = 0.008, *η*_*p*_^2^ = 0.17), and a significant main effect of Congruency Level of Preceding Trial (*F*(1, 31) = 9.62, *p* = 0.002, *η*_*p*_^2^ = 0.24). The analysis also revealed a significant CSE (*F*(1, 31) = 6.68, *p* = 0.007, *η*_*p*_^2^ = 0.18). There was no three-way interaction involving Congruency Level of Current Trial, Congruency Level of Preceding Trial, and Experiment (*F*(1, 31) = 0.37, *p* = 0.27, *η*_*p*_^2^ = 0.01). No other effect reached significance (*F’s* < 2).

An analysis involving category alternation trials, the congruency effect reached significance (*F*(1, 31) = 15.47, *p* < 0.001, *η*_*p*_^2^ = 0.33) as well as the main effect of Congruency Level of Preceding Trial (*F*(1, 31) = 13.31, *p* < 0.001, *η*_*p*_^2^ = 0.30). The two-way interaction between Experiment and Congruency Level of Current Trial reached significance (*F*(1, 31) = 8.21, *p* = 0.0037, *η*_*p*_^2^ = 0.21) as well as the three-way interaction of CSE and Experiment reached significance (*F*(1, 31) = 4.42, *p* = 0.019, *η*_*p*_^2^ = 0.12). No other effects reached significance (all *F’s* < 1).

***ER.*** The overall ANOVA yielded a significant congruency effect (*F*(1, 31) = 3.91, *p* = 0.029, *η*_*p*_^2^ = 0.11), and a significant three-way interaction involving the CSE and Sequence of Target/Distractor Category (*F*(1, 31) = 4.78, *p* = 0.018, *η*_*p*_^2^ = 0.13). No other effect reached significance (all *F’s* < 1). For every result regarding the overall ANOVA, see Appendix C.

For category-order repetition trials, there was a significant congruency effect (*F*(1, 31) = 2.98, *p* = 0.047, *η*_*p*_^2^ = 0.09). More importantly, the CSE reached significance (*F*(1, 31) = 5.07, *p* = 0.016, *η*_*p*_^2^ = 0.14) which was not modulated by Experiment (*F*(1, 31) = 0.24, *p* = 0.31, *η*_*p*_^2^ = 0.01). No other effect reached significance (all *F’s* < 1).

For category alternation trials, the CSE did not reach significance (*F*(1, 31) = 0.04, *p* = 0.42, *η*_*p*_^2^ = 0.00). Again, there was no modulation by Experiment (*F*(1, 31) = 0.41, *p* = 0.26, *η*_*p*_^2^ = 0.01). The other effects did not reach significance as well (all *F’s* < 2).

### Discussion

The overall congruency effect was markedly smaller than in Experiment 1A, suggesting that participants used stimulus color for target-distractor discrimination. Notwithstanding this reduction, the CSE was significant in RTs, and there was no three-way interaction with the sequence of category order. As confirmed by a significant four-way interaction in a combined analysis of the two experiments, this lacking influence of category-order sequence on the CSE differed from Experiment 1. This pattern of results was expected on the assumption that adding a redundant feature discriminating targets and distractors would result in a control strategy based on stimulus information other than stimulus category. Given the complete confound of temporal position and stimulus color, we cannot determine which of these two features attentional selection was based on, however.

As the CSE was not larger in category repetition trials than in category alternation trials, our results provide no evidence that category-based control was applied at all. The application of category-based control would thus not seem to be an obligatory consequence of the long temporal separation of the presentation of target and distractor stimuli. We acknowledge, however, that firm conclusions regarding this issue have to await future investigations recruiting larger sample sizes. In particular, the null effect concerning the difference in the size of the CSE in category repetition trials and category alternation trials should be considered with caution.

## General discussion

The current study investigated trial-to-trial attentional adjustment in a priming protocol, in which targets and distractors could be discriminated by multiple features. Whereas one or two features (i.e., temporal position within a trial and, in Experiment 2, stimulus color) maintained a constant assignment to targets and distractors throughout the experimental session, targets and distractors also differed with regard to their semantic category (i.e., letters and digits) but the assignment of these categories to the target and the distractor was chosen randomly on each trial. This arrangement seems suitable to provide evidence for the concurrent usage of attentional control strategies based on the constant and on the variable features. This is because control based on the constant feature(s) predicts a CSE in both category repetition and category alternation trials. In contrast, control based on the variable feature predicts a CSE in category repetition trials but not in category alternation trials. Observing a larger CSE in category repetition trials than in category alternation trials thus points to the occurrence of both strategies. This pattern of results was observed in previous experiments involving a temporal flanker task with a short distractor-target SOA (Tomat et al. 2021).

Contrasting with these previous findings, administering a longer SOA (i.e., 1000 ms or 1200 ms) yielded result patterns consistent with using a single control strategy. More precisely, the CSE was confined to category repetition trials in Experiments 1A and 1B. In contrast, there was no indication of a difference in the strengths of the CSE in Experiment 2, in which we added a stimulus feature (i.e., color) that redundantly distinguished targets and distractors. Together, these results can e conceived of as preliminary evidence that the effectiveness of attentional strategies applied in parallel or the choice of a particular attentional strategy in priming tasks depends on contextual variables discriminating targets and distractors, such as the length of the time interval between their onsets or the presence of an additional discriminating feature. Empirically, more research is needed to provide a systematic overview of determinants that yield evidence for particular control strategies, such as temporal order control.

At the theoretical level, we would like to emphasize that failing to obtain such evidence—as in the results of Experiments 1A and 1B—does not per se rule out that temporal order control was applied. This is because, for one thing, it is conceivable that temporal order control is applied in parallel with another control measure, going undetected if it is evened out in trials in which the two measures have opposite directions. For another, it might be conjectured that temporal order control is sensitive to the occurrence of particular context features, such as the presentation order of stimulus categories, resulting in the absence of control effects when this context changes. In line with this reasoning, the absence of the CSE under conditions of contextual changes has previously been attributed to episodic retrieval of the control set, the likelihood of which is affected by contextual correspondence (e.g., Dignath et al. 2019; Spapé and Hommel, 2008). Arguably, accounts that attribute the CSE to the expectation of repetition of the previous congruency level (e.g., Duthoo et al. 2013; Erb and Aschenbrenner 2019; Jiménez and Méndez 2013, 2014) would also be consistent with such context-dependency if it is assumed that expecting the repetition of the congruency level is bound to expecting the repetition of the context factor.

Contextual features distinguishing target and distractor stimuli may thus pose particular interpretation problems as findings of selective occurrence of the CSE in context repetition trials seem equally well explained by assuming context-dependent temporal order control or by control based on the contextual feature. Noteworthy, however, context-dependent (temporal order) control does not seem to predict a reversal of the CSE in trials associated with the reversal of the assignment of the values of the contextual feature to the target and the distractor, as we observed (marginally significant) in Experiment 1 of the current study. The same reasoning can be applied to another account of the CSE, hitherto unmentioned in this article, which has been dubbed temporal learning (e.g., Schmidt 2013).^3^

Against this background, the general experimental setup used in the current study (i.e., combining features with constant and with variable assignment to targets and distractors) may provide a useful tool in the search for factors affecting a shift between the strengths of concurrently applied control strategies by yielding results patterns ranging from indication of exclusive reliance of control based on the “constant feature” (i.e., a CSE of equal strength in repetition and alternation trials) to indication of dominant or exclusive reliance on the “variable feature” (i.e., a CSE in repetition trials alongside with a reversed CSE in alternation trials).

Another contextual factor possibly influencing the application of a particular control strategy is the amount of practice with a given task. Based on findings of a CSE in a Stroop task selectively in the first trial blocks of an experimental session, Mayr and Awh (2009) suggested that trial-to-trial adjustment of attention is abandoned once a certain degree of task practice has been reached. To investigate alterations in the application of attentional strategies during short-term practice, we reanalyzed all experiments of the current study by comparing performance during the first and the second half of the experimental session. The results of these reanalyses are displayed in the appendix (i.e., Appendix D to L). Although these results should be considered with caution because the number of trials per condition is very low for some conditions (i.e., congruent-to-congruent transitions), they do suggest some systematic influence of task practice. Specifically, regarding RTs, the difference of the strength of the CSE in category repetition trials and category alternation trials in Experiment 1A and 1B (i.e., the occurrence of a CSE in repetition trials but not in alternation trials) tended to be larger in the first half than in the second half of the experimental session. In contrast, in Experiment 2, the CSE in alternation trial (i.e., our primary indication of temporal order control) occurred selectively during the second half of the experimental session. Although these results do not confirm Mayr and Awh’s (2009) conjecture, they appear in line with the notion of practice-related changes in attentional strategies under conditions of availability of multiple features upon which attentional control can be based upon.^4^

Although our primary interest concerned the investigation of the CSE, another type of sequential processing adjustment, dubbed *post-conflict slowing,* has been discussed (e.g., Erb and Marcovitch 2019; Erb et al. 2019; Verguts et al. 2011; Wendt et al. 2006). Studies with reaching responses consistently demonstrated that the effect can be observed in response initiation times, whereas it was absent in movement times or the curvature of the response trajectory (e.g., Erb and Marcovitch 2019; Erb et al. 2019), suggesting a conflict-induced adjustment of the response threshold. In line with previous temporal flanker task experiments, in which post-conflict slowing was occasionally observed, RTs were longer after incongruent than after congruent trials in Experiment 1A and Experiment 2 of the current study. In contrast, no comparable effect occurred in Experiment 1B. In a reanalysis of the combined RT data of Experiment 1A and 1B, the two-way interaction of Congruency on the Preceding Trial and Experiment reached statistical significance (*F*(1, 45) = 4.45, *p* < 0.05). Although we can only speculate about the cause of this discrepancy we note that, in general, post-conflict slowing appears to be a less robust phenomenon than the CSE. An obvious possible reason for this is that, when both adjustments (i.e., attentional adjustment and adjustment of the response threshold) take place, post-conflict slowing tends to be masked by an opposing tendency (i.e., speed-up) in incongruent trials following incongruent trials (Verguts et al. 2011). Theoretical progress concerning the interplay of the two mechanisms clearly depends on determining experimental factors crucial for the occurrence of each of the two effects. Applying procedures in which the CSE is confined to a subset of otherwise similar conditions (such as category repetition trials vs. category alternation trials in Experiments 1A and 1B) may prove helpful in this regard.

In summary, extending previous evidence for temporal order control in priming tasks (Dignath et al. 2021; Tomat et al. 2021), the results of the current study suggest that contextual variations concerning, for instance, the length of the distractor-target SOA or the availability of a redundant perceptual feature discriminating task-relevant from task-irrelevant information, affects the strengths or even the choice of control strategies applied in that particular context. Examining the CSE in trials associated with repetition vs. alternation of the assignment of a contextual feature to targets and distractors may play a major role in identifying determinants underlying these dependencies.

## Figures and Tables

**Fig. 7:**
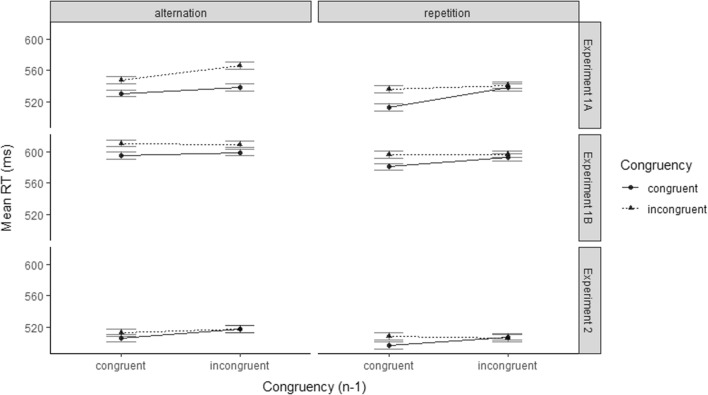
Mean RTs of temporal flanker task trials in Experiment 1A (top), 1B (middle), and Experiment 2 (bottom). Data are shown as a function of Congruency Level of Current Trial (congruent vs. incongruent), Congruency Level of Preceding Trial (congruent_*n*−1_ vs. incongruent_*n*−1_), Sequence of Target/Distractor Category (alternation vs. repetition), and Experiment (1A vs. 1B vs. 2)

**Fig. 8:**
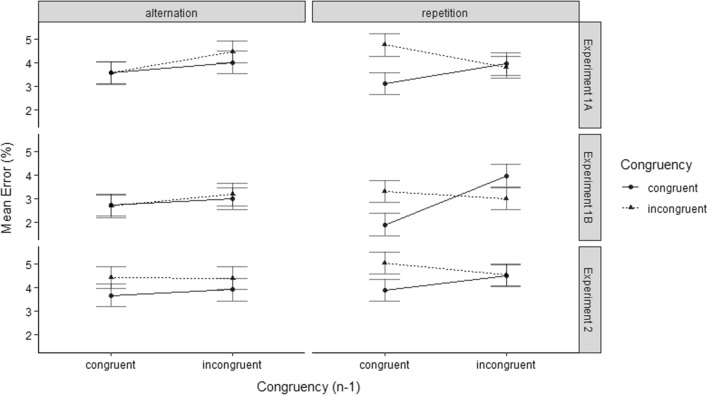
Mean ERs of temporal flanker task trials in Experiment 1A (top), 1B (middle), and Experiment 2 (bottom). Data are shown as a function of Congruency Level of Current Trial (congruent vs. incongruent), Congruency Level of Preceding Trial (congruent_*n*−1_ vs. incongruent_*n*−1_), Sequence of Target/Distractor Category (alternation vs. repetition), and Experiment (1A vs. 1B vs. 2)

**Fig. 9:**
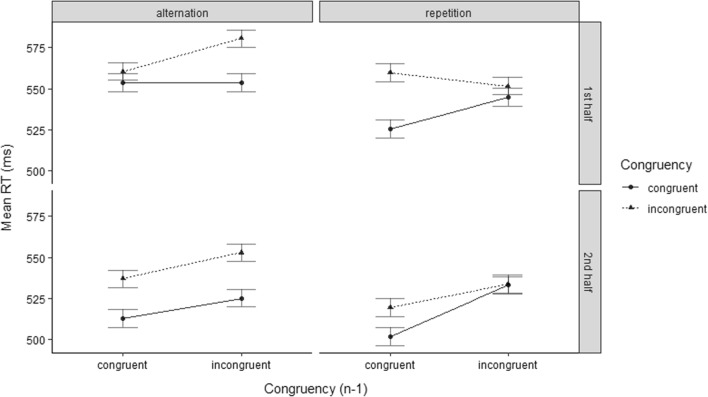
Mean RTs of temporal flanker task trials in Experiment 1A. Data are shown as a function of Congruency Level of Current Trial (congruent vs. incongruent), Congruency Level of Preceding Trial (congruent_*n*−1_ vs. incongruent_*n*−1_), Sequence of Target/Distractor Category (alternation vs. repetition), and Half of Experiment (1st vs. 2nd)

**Fig. 10:**
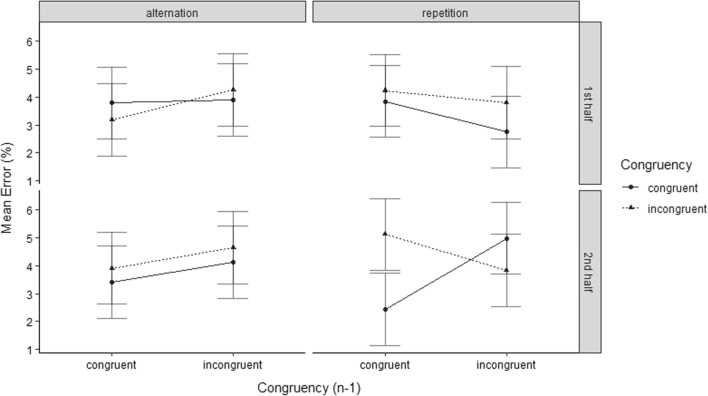
Mean ERs of temporal flanker task trials in Experiment 1A. Data are shown as a function of Congruency Level of Current Trial (congruent vs. incongruent), Congruency Level of Preceding Trial (congruent_*n*−1_ vs. incongruent_*n*−1_), Sequence of Target/Distractor Category (alternation vs. repetition), and Half of Experiment (1st vs. 2nd)

**Fig. 11:**
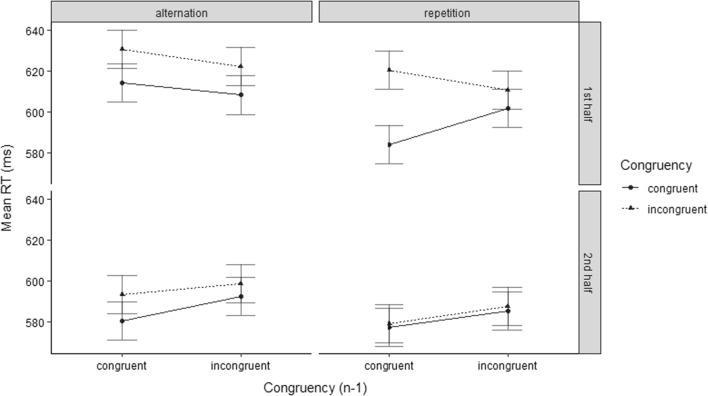
Mean RTs of temporal flanker task trials in Experiment 1B. Data are shown as a function of Congruency Level of Current Trial (congruent vs. incongruent), Congruency Level of Preceding Trial (congruent_*n*−1_ vs. incongruent_*n*−1_), Sequence of Target/Distractor Category (alternation vs. repetition), and Half of Experiment (1st vs. 2nd)

**Fig. 12:**
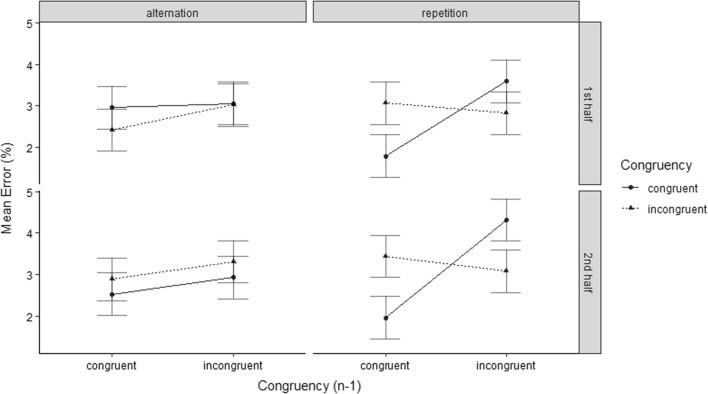
Mean error proportions of temporal flanker task trials in Experiment 1B. Data are shown as a function of Congruency Level of Current Trial (congruent vs. incongruent), Congruency Level of Preceding Trial (congruent_*n*−1_ vs. incongruent_*n*−1_), Sequence of Target/Distractor Category (alternation vs. repetition), and Half of Experiment (1st vs. 2nd)

**Fig. 13:**
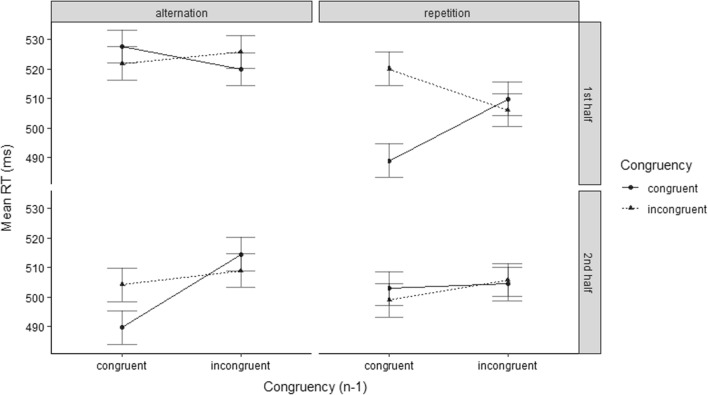
Mean RTs of temporal flanker task trials in Experiment 2. Data are shown as a function of Congruency Level of Current Trial (congruent vs. incongruent), Congruency Level of Preceding Trial (congruent_*n*−1_ vs. incongruent_*n*−1_), Sequence of Target/Distractor Category (alternation vs. repetition), and Half of Experiment (1st vs. 2nd)

**Fig. 14:**
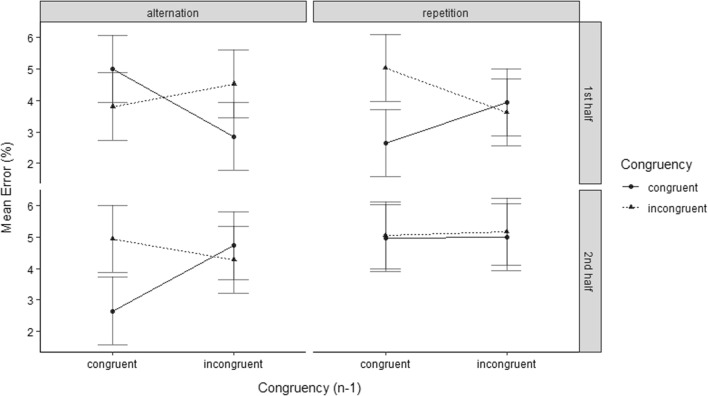
Mean ERs of temporal flanker task trials in Experiment 2. Data are shown as a function of Congruency Level of Current Trial (congruent vs. incongruent), Congruency Level of Preceding Trial (congruent_*n*−1_ vs. incongruent_*n*−1_), Sequence of Target/Distractor Category (alternation vs. repetition), and Half of Experiment (1st vs. 2nd)

## Data Availability

The data and materials for all experiments reported here are available from the corresponding author upon reasonable request. None of the experiments was preregistered.

## Appendix A

Plotted mean RTs of Experiment 1A, Experiment 1B, and Experiment 2 with the same scaling (Fig. 7).

## Appendix B

Plotted mean ERs of Experiment 1A, Experiment 1B, and Experiment 2 with the same scaling (Fig. 8)

## Appendix C

Table of results for the overall analysis of the comparison between Experiment 1A and 2 (Table 1).

**Table 1:** ANOVA results for RTs (and for the mean error proportions in parentheses) of the Comparison between Experiment 1A and Experiment 2

Predictor	*df*_Num_	*df*_Den_	*F*	*p*	η_p_^2^
Experiment (E)	1	31	2.30 (0.32)	0.070 (0.29)	0.07 (0.01)
Congruency Level of Current Trial (C)	1	31	12.67 (3.91)	< 0.001 (0.028)	0.3 (0.11)
Congruency Level of Preceding Trial (C_*n*−1_)	1	31	21.52 (0.67)	< 0.001 (0.21)	0.1 (0.00)
Sequence of Target/Distractor Category (S)	1	31	32.59 (0.95)	< 0.001 (0.17)	0.4 (0.02)
E x C	1	31	4.73 (0.05)	0.019 (0.42)	0.01 (0.01)
E x C_*n*−1_	1	31	3.18 (0.20)	0.042 (0.33)	0.5 (0.03)
E x S	1	31	1.48 (0.98)	0.12 (0.16)	0.05 (0.03)
C x C_*n*−1_	1	31	2.69 (2.07)	0.056 (0.08)	0.08 (0.06)
C x S	1	31	2.26 (0.44)	0.071 (0.25)	0.01 (0.00)
C_*n*−1_ × S	1	31	0.00 (0.91)	0.49 (0.17)	0.07 (0.01)
E x C x C_*n*−1_	1	31	0.30 (0.00)	0.29 (0.49)	0.1 (0.01)
E x C x S	1	31	3.93 (0.47)	0.028 (0.25)	0.00 (0.03)
E x C_*n*−1_ × S	1	31	0.52 (0.67)	0.24 (0.21)	0.02 (0.02)
C x C_*n*−1_ × S	1	31	8.79 (4.78)	0.003 (0.02)	0.2 (0.13)
E x C x C_*n*−1_ × S	1	31	3.86 (0.85)	0.029 (0.18)	0.1 (0.03)

## Appendix D

Plotted mean RTs of reanalysis of Experiment 1A (Fig. 9)

## Appendix E

Plotted mean ERs of reanalysis of Experiment 1A (Fig. 10)

## Appendix F

Table of results of Reanalysis of Experiment 1A (Table 2).

**Table 2:** ANOVA results for mean RTs (and for the mean ERs in parentheses) of Reanalysis of Experiment 1A

Predictor	*df*_Num_	*df*_Den_	*F*	*P*	η_p_^2^
Congruency Level of Current Trial (C)	1	16	10.16 (1.47)	0.003 (0.12)	0.39 (0.08)
Congruency Level of Preceding Trial (C_*n*−1_)	1	16	15.01 (0.66)	< 0.001 (0.22)	0.48 (0.04)
Sequence of Target/Distractor Category (S)	1	16	14.34 (0.01)	0.001 (0.46)	0.47 (0.00)
Half of Experiment (H)	1	16	20.40 (2.38)	< 0.001 (0.07)	0.56 (0.13)
C x C_*n*−1_	1	16	0.53 (0.60)	0.24 (0.23)	0.03 (0.04)
C x S	1	16	1.99 (0.92)	0.09 (0.18)	0.11 (0.05)
C_*n*−1_ × S	1	16	0.10 (1.49)	0.38 (0.12)	0.01 (0.09)
C x H	1	16	0.08 (0.27)	0.39 (0.31)	0.01 (0.02)
C_*n*−1_ × H	1	16	4.54 (2.71)	0.025 (0.06)	0.22 (0.14)
S x H	1	16	1.60 (0.15)	0.11 (0.35)	0.09 (0.01)
C x C_*n*−1_ × S	1	16	13.13 (4.1)	0.001 (0.03)	0.45 (0.2)
C x C_*n*−1_ × H	1	16	0.05 (3.95)	0.41 (0.03)	0.00 (0.20)
C x S x H	1	16	4.31 (0.11)	0.027 (0.38)	0.21 (0.01)
C_*n*−1_ × S x H	1	16	1.88 (0.85)	0.095 (0.19)	0.11 (0.05)
C x C_*n*−1_ × S x H	1	16	3.50 (1.06)	0.040 (0.16)	0.18 (0.06)

## Appendix G

Plotted RTs of reanalysis of Experiment 1B (Fig. 11)

## Appendix H

Plotted error proportions of reanalysis of Experiment 1B (Fig. 12).

## Appendix I

Table of results of Reanalysis of Experiment 1B (Table 3).

**Table 3:** ANOVA results for RTs (and for the mean error proportions in parentheses) of Reanalysis of Experiment 1B

Predictor	*df*_Num_	*df*_Den_	*F*	*P*	η_p_^2^
Congruency Level of Current Trial (C)	1	29	14.69 (0.38)	< 0.001 (0.27)	0.34 (0.01)
Congruency Level of Preceding Trial (C_*n*−1_)	1	29	1.27 (14.16)	0.13 < (0.01)	0.04 (0.32)
Sequence of Target/Distractor Category (S)	1	29	23.93 (0.32)	< 0.001 (0.29)	0.45 (0.01)
Half of Experiment (H)	1	29	10.36 (0.82)	0.002 (0.19)	0.26 (0.03)
C x C_*n*−1_	1	29	2.33 (8.59)	0.069 (0.003)	0.07 (0.23)
C x S	1	29	0.00 (0.10)	0.49 (0.37)	0.00 (0.00)
C_*n*−1_ × S	1	29	1.81 (1.30)	0.094 (0.13)	0.06 (0.04)
C x H	1	29	9.28 (0.51)	0.002 (0.24)	0.24 (0.02)
C_*n*−1_ × H	1	29	1.82 (0.11)	0.094 (0.37)	0.06 (0.00)
S x H	1	29	1.46 (0.63)	0.12 (0.22)	0.05 (0.02)
C x C_*n*−1_ × S	1	29	1.15 (9.37)	0.15 (0.002)	0.04 (0.24)
C x C_*n*−1_ × H	1	29	0.92 (0.60)	0.17 (0.22)	0.03 (0.02)
C x S x H	1	29	3.20 (1.01)	0.04 (0.16)	0.10 (0.03)
C_*n*−1_ × S x H	1	29	0.68 (0.05)	0.21 (0.41)	0.02 (0.00)
C x C_*n*−1_ × S x H	1	29	1.50 (0.02)	0.11 (0.44)	0.05 (0.00)

## Appendix J

Plotted RTs of reanalysis of Experiment 2 (Fig. 13)

## Appendix K

Plotted mean ERs of reanalysis of Experiment 2 (Fig. 14).

## Appendix L

Table of results of Reanalysis of Experiment 2 (Table 4).

**Table 4:** ANOVA results for RTs (and for the mean ERs in parentheses) of Reanalysis of Experiment 2

Predictor	*df*_Num_	*df*_Den_	*F*	*p*	η_p_^2^
Congruency Level of Current Trial (C)	1	15	3.23 (2.07)	0.046 (0.08)	0.18 (0.12)
Congruency Level of Preceding Trial (C_*n*−1_)	1	15	4.08 (< 0.001)	0.031 (0.50)	0.21 (0.00)
Sequence of Target/Distractor Category (S)	1	15	38.22 (1.17)	< 0.001 (0.15)	0.72 (0.07)
Half of Experiment (H)	1	15	5.79 (3.77)	0.015 (0.04)	0.28 (0.20)
C x C_*n*−1_	1	15	4.44 (1.09)	0.026 (0.16)	0.23 (0.07)
C x S	1	15	1.65 (< 0.001)	0.11 (0.50)	0.10 (0.00)
C_*n*−1_ × S	1	15	0.33 (< 0.001)	0.29 (0.50)	0.02 (0.00)
C x H	1	15	0.61 (0.02)	0.22 (0.45)	0.04 (0.00)
C_*n*−1_ × H	1	15	2.34 (0.90)	0.07 (0.18)	0.14 (0.06)
S x H	1	15	16.77 (3.69)	< 0.001 (0.04)	0.53 (0.20)
C x C_*n*−1_ × S	1	15	1.54 (1.52)	0.12 (0.12)	0.09 (0.09)
C x C_*n*−1_ × H	1	15	0.16 (0.69)	0.35 (0.21)	0.01 (0.04)
C x S x H	1	15	3.59 (2.18)	0.039 (0.04)	0.19 (0.13)
C_*n*−1_ × S x H	1	15	4.88 (5.87)	0.021 (0.23)	0.25 (0.04)
C x C_*n*−1_ × S x H	1	15	22.62 (8.71)	< 0.001 (0.005)	0.60 (0.37)

## Footnotes

1 Reduction of the congruency effect when the distractor-target SOA is increased is a typical finding (e.g., Gillich et al. 2019; Machado et al. 2013).

2 In fact, this is a common method for priming tasks employed in investigations of the CSE. Various features have been added to discriminate targets and distractors, such as stimulus size (e.g., Weissman et al. 2015; or number of copies, e.g., Schmidt and Weissman 2014).

3 According to Schmidt’s (2013) temporal learning account, performance is facilitated if the preceding trial’s response timing (i.e., the time needed to come to a response decision) matches the current trial’s response timing. Given that responding is generally faster in congruent than in incongruent trials, the CSE might thus be brought about by better matches of the preceding trial’s response timing with the subsequent trial’s response timing when the congruency level repeats.

4 We are grateful to Luis Jiménez for drawing our attention to this possibility.
